# Synthesis, Structural Characterization and Biological Activity Evaluation of Novel Cu(II) Complexes with 3-(trifluoromethyl)phenylthiourea Derivatives

**DOI:** 10.3390/ijms232415694

**Published:** 2022-12-10

**Authors:** Aleksandra Drzewiecka-Antonik, Marta Struga, Agnieszka Głogowska, Ewa Augustynowicz-Kopec, Katarzyna Dobrzyńska, Alicja Chrzanowska, Anna Wolska, Paweł Rejmak, Marcin T. Klepka, Małgorzata Wrzosek, Anna Bielenica

**Affiliations:** 1 Institute of Physics, Polish Academy of Sciences, Al. Lotnikow 32/46, PL-02668 Warsaw, Poland; 2 Chair and Department of Biochemistry, Medical University of Warsaw, Banacha 1, PL-02097 Warsaw, Poland; 3 Institute of Tuberculosis and Lung Diseases, Microbiology Department, Plocka 26, PL-01138 Warsaw, Poland; 4 Department of Pharmaceutical Microbiology, Faculty of Pharmacy, Warsaw Medical University, 3 Oczki Street, PL-02007 Warsaw, Poland; 5 Department of Biochemistry and Pharmacogenomics, Faculty of Pharmacy, Medical University of Warsaw, Banacha 1, PL-02097 Warsaw, Poland

**Keywords:** Cu(II) complexes, thiourea, antimicrobial activity, ATR-IR, UV-Vis, XAFS

## Abstract

Copper complexes with 1,3-disubstituted thiourea derivatives, all containing 3-(trifluoromethyl)phenyl tail and 1-alkyl/halogen-phenyl substituent, were synthesized. The experimental spectroscopic studies and theoretical calculation revealed that two ligands coordinate to Cu(II) in a bidentate fashion via thiocarbonyl S and deprotonated N atoms of thiourea moiety. Such monomers are characteristic of alkylphenylthiourea complexes, whereas the formation of a sandwich-type dimer is observed for halogeno derivatives. For the first time, the structural identifications of CuN_2_S_2_-based complexes using experimental and theoretical X-ray absorption near edge structure are demonstrated. The dimeric halogeno derivatives showed higher antimicrobial activity in comparison with alkylphenylthiourea complexes. The Cu(II) complex of 1-(4-chloro-3-nitrophenyl)-3-[3-(trifluoromethyl)phenyl]thiourea was active against 19 strains of methicillin-resistant *Staphylococci* (MIC = 2 µg/mL). This derivative acted as a dual inhibitor of DNA gyrase and topoisomerase IV isolated from *Staphylococcus aureus*. Additionally, complexes of halogenphenylthiourea strongly inhibited the growth of mycobacteria isolated from tuberculosis patients, even fourfold stronger than the reference isoniazid. The complexes exerted weak to moderate antitumor activity (towards SW480, SW620, and PC3) being non-toxic towards normal HaCaT cells.

## 1. Introduction

Thiourea derivatives are widely studied and applied as crucial reagents in organic synthesis, and as bioactive agents and drugs in medicine [1,2]. There is also a growing interest in pharmaceutical applications of metal complexes with thiourea ligands, notably copper coordination compounds [3,4,5]. Regarding copper complexes with ligands containing thiourea moiety, the ones containing thiosemicarbazone (NH_2_ = NH-C(=S)-NH_2_) or thiosemicarbazide (NH_2_-NH-C(=S)-NH_2_) derivatives have been the most widely studied for their use as anticancer chemotherapeutics [6,7], antimicrobial drugs [5,8], neuroprotective agent in Alzheimer’s disease [9,10], and radiopharmaceuticals [11,12]. The broad spectrum of bioactivity of these compounds can be correlated with copper’s ability to participate in redox reactions, and to coordinate ligands in 3D configuration, thus enabling interaction with particular molecular targets. The thiourea moiety can bind Cu cations in several ways, e.g., act as monodentate ligands via S atom in mononuclear complexes [6,13,14], bind to metal ions forming *S*-bridges in dinuclear complexes [6,11,14], or chelate by *S,N* atoms [7,11,14].

We have noticed that relatively few publications focus on bioactive copper complexes with thiourea-based ligands without N-N bonds (such as in thiosemicarbazides and thiosemicarbazones), especially with *N*,*N*-disubstituted thioureas [15]. Therefore, in recent years, several series of copper(II) complexes with *N*,*N*-disubstituted thiourea derivatives have been designed, synthesized, and characterized by our group. The obtained compounds possess high to moderate antibacterial activity [16,17] and some of them show strong anticancer potential against colon and prostate cancer [18,19]. Among these compounds, the complex with 1-1-(3,4-dichlorophenyl)-3-[3-(trifluoromethyl) phenyl]thiourea exerts the highest activity against 30 clinically isolated strains of *S. aureus* and *S. epidermidis* (MIC 0.5–2 μg/mL). Moreover, copper (II) complexes with 3-(4-chloro-3-nitrophenyl)thiourea were cytotoxic to the tumor cells lines: SW480, SW620, and PC3 in the low micromolar range, without affecting the normal cells. Due to this significant biological activity (stronger than for the parent ligand and reference drug), we decided to design further connections with copper based on the ligands of the same family. In this work, we present the synthesis and characterization of five new complexes with 3-(trifluoromethyl)phenylthiourea derivatives. The new compounds were evaluated for their antimicrobial potency including (*i*) the investigation toward standard bacterial and fungi strains; (*ii*) tests on methicillin-resistant *Staphylococcus aureus* (MRSA) and *Staphylococcus epidermidis* (MRSE) clinical strains; and as it is the first time for those series, (*iii*) experiments on standard *M. tuberculosis* strain H_37_Rv and two “wild” strains isolated from patients suffering from tuberculosis. Moreover, the mechanism of the antibacterial action of the most active complex was determined. In parallel, new complexes were evaluated for their cytotoxicity on the colon and prostate cancer (SW480, SW620, and PC3) cell lines. 

To analyze the impact of the structure of new compounds on their bioactivity, the key is to determine the molecular structure of the studied complexes. Since we were not able to obtain good-quality crystals of the discussed compounds, we determined the molecular structure of the complexes in their powder form. For this purpose, coordination compounds were studied by elemental analysis, ATR-IR (Attenuated total reflection infrared), and UV–visible spectroscopies. Moreover, XANES (X-ray Absorption Near Edge Structure) and EXAFS (Extended X-ray Absorption Fine Structure) experiments were performed using synchrotron radiation. Data obtained from XAFS (X-ray absorption fine structure) analysis combined with the density functional theory (DFT) and XANES calculations allowed us to propose a 3D molecular structure of novel complexes. 

## 2. Results and Discussion

### 2.1. Synthesis

**Figure 1:**
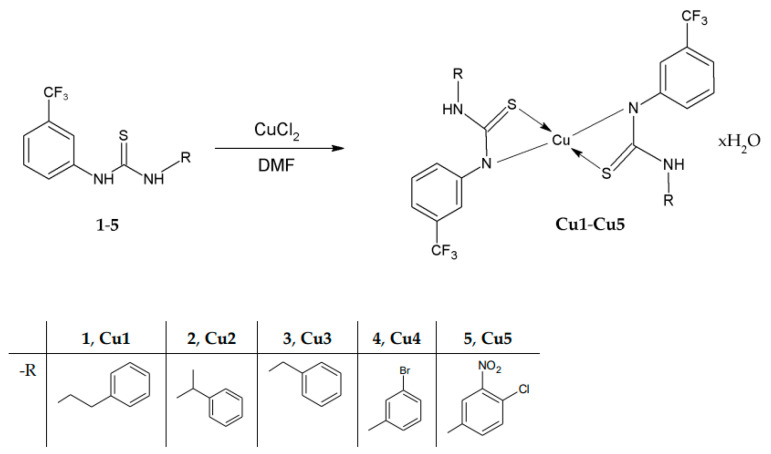
Synthesis path of **Cu1**–**Cu5** complexes with the following formulas: Cu(1)_2·_H_2_O, Cu(2)_2_·0.75H_2_O, Cu(3)_2_·0.5H_2_O, Cu(4)_2_·0.5H_2_O, Cu(5)_2_·H_2_O. DMF—dimethylformamide.

Copper-based thiourea complexes **Cu1**–**Cu5** were synthesized by the reaction of CuCl_2_ with 3-(trifluoromethyl)phenylthiourea derivatives (**1**–**5**) (Figure 1). The synthesis and characterization of initial ligands **1**, **3**, **4** [20], **2** [21], and **5 [22]** have been described elsewhere. The compounds chosen for complexation reaction came from the group of alkylphenyl (ligands **1**–**3**) and halogenphenyl (ligands **4**, **5**) thiourea derivatives. Their complexation with copper gave compounds in the form of microcrystalline powder. The elemental analysis of **Cu1**–**Cu5** determined the metal-to-ligand ratio as 1:2 and indicated the presence of water molecules in the molecular structure of new compounds.

### 2.2. Spectroscopic Characterization

The complexation reactions were followed by the infrared and UV-Vis spectroscopies. Results of the analysis of IR and UV-Vis spectra of complexes revealed the oxidation state of copper, symmetry of the metal–ligand interactions, and indicated the atoms coordinating to the metallic center. The comparative XANES analysis confirmed the oxidation state of the metallic center, whereas EXAFS analysis gave us information about the radial distributions of atoms around Cu(II). These experimental studies completed with DFT and XANES calculations allowed us to build and optimize structural models of new coordination compounds. The described studies are presented below.

#### 2.2.1. ATR-IR Studies

The attenuated total reflection Fourier transform-infrared spectra of five copper(II) complexes with 1,3-disubstitued thioureas (**Cu1**–**Cu5**, Figure 1) and their parent ligands (**1**–**5**, Figure 1) were recorded over the range 4000–400 cm^−1^. The fingerprint regions (1000–400 cm^−1^) have been compared for each pair of the organic ligand–Cu(II) complex. The notable differences in this part of the spectra confirmed that new compounds (**Cu1**–**Cu5**) have been formed. 

**Figure 2:**
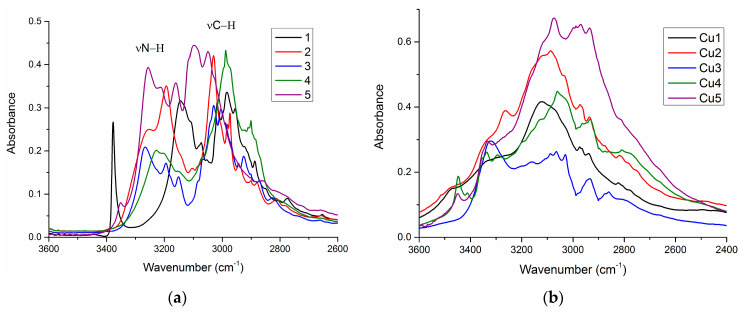
ATR-IR spectra of (**a**) ligands in the region 3600–2600 cm^−1^ (**b**) and their Cu(II) complexes in the region 3600–2400 cm^−1^.

The region between 3400 and 2800 cm^−1^ in organic ligand spectra contains two broad features with several maxima corresponding to the N–H and C–H stretching vibrations (Figure 2a). The high intensity of these bands is due to the presence of hydrogen bonding between C/N–H groups and electronegative atoms such as S, N, Cl, and Br. In the spectra of the copper(II) complexes (Figure 2b), these two broad features merge into one wide band, extending from 3500 to 2600 cm^−1^. This widening indicates the presence of water molecules in **Cu1**–**Cu5**, which is consistent with the results of the elemental analysis. Moreover, a decrease in the intensity of the bands >3200 cm^−1^ and their extension are observed in the high-energy part of the spectra of complex **Cu1**–**Cu5**. This confirms the partial deprotonation of the N–H groups of the ligand molecules as a result of the complexation reaction. 

The thiourea moiety in all compounds yields two absorption bands corresponding to the C = S stretching vibrations (Table 1). These bands are observed in the regions 1351–1342 cm^−1^ and 858–844 cm^−1^ in spectra of the organic ligands (**1**–**5**). After complexation, their position was shifted to the lower values, around 1300 cm^−1^ and 764–754 cm^−1^ (Table 1), respectively, proving the coordination of the sulfur atom of the ligands [23,24]. 

**Figure 3:**
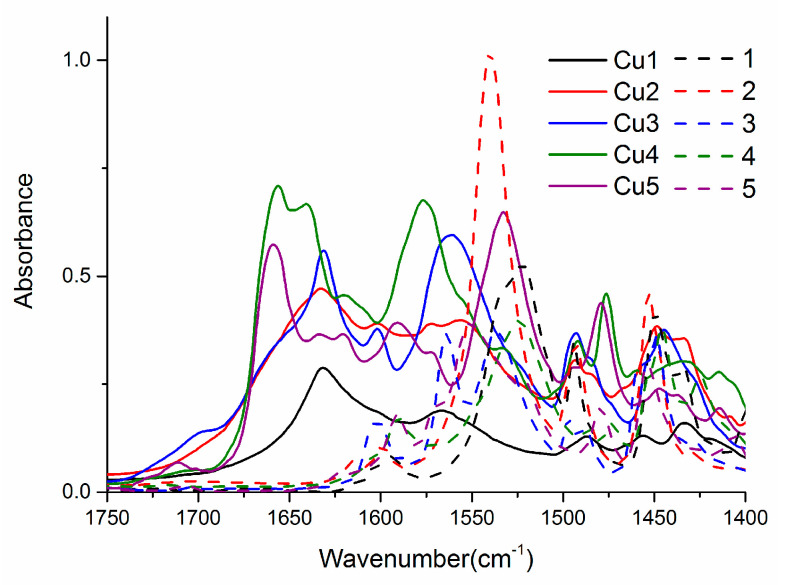
ATR-IR spectra of complexes and free ligands in the region 1750–1400 cm^−1^.

Moreover, the several bands in the region of 1600–1400 cm^−1^ (Figure 3) in free ligands as well as their Cu(II) complexes indicate the presence of C–C and C–N bonds within their molecules. Interestingly, in the spectra of **Cu1**–**Cu5**, this range is widened to 1700 cm^−1^. This bandwidth extension was observed for the previous series of analogous complexes [17]. The calculated harmonics using DFT revealed that this is a consequence of increased intensities of antisymmetric ring C–C stretching modes within the molecules of complexes.

#### 2.2.2. UV-Vis Analysis

**Figure 4:**
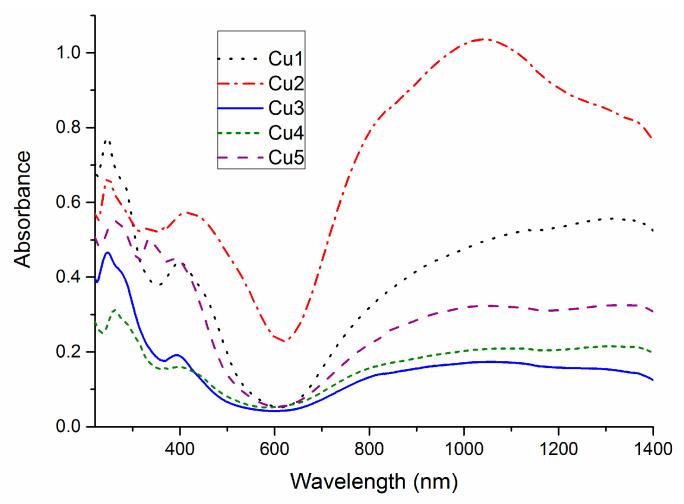
UV-Vis spectra in the range of 220–1400 nm for complexes **Cu1–Cu5**.

The electronic spectra of thiourea complexes (**Cu1**–**Cu5**) are presented in Figure 4. Around 250 nm there is a strong band corresponding to the transition of the organic part of molecules. A ligand-to-metal charge transfer (LMCT) band is found in the region of 400–420 nm. This band is tailing into 600 nm, which allows us to assign it to S→Cu(II) transition within the complexes [18,23,24]. The broad feature between 700 and 1400 nm corresponds to d-d transitions. Within the studied complexes, there are notable differences in the intensities of d-d bands (Figure 4), which are correlated with differences in the geometry around the metal cation. The low intensity of the d-d band indicates that copper cations are coordinated in a centrosymmetric fashion, whereas the higher intensity suggests the noncentrosymmetric geometry [18].

#### 2.2.3. XANES Comparative Analysis

**Figure 5:**
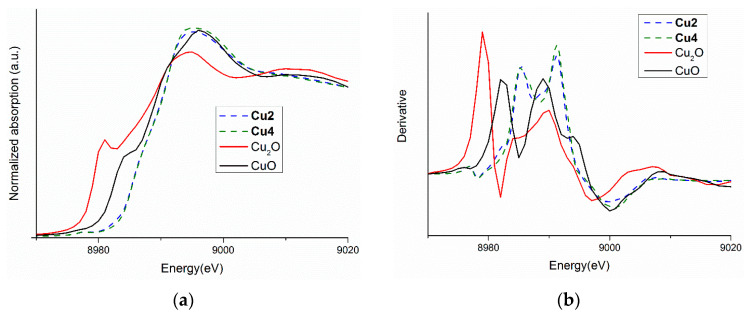
(**a**) Cu K-edge spectra of two complexes **Cu2**, **Cu4**, and two reference oxides: Cu_2_O, CuO, and (**b**) their first derivatives.

The copper oxidation state in the synthesized compounds was determined by XANES spectra comparative analysis. Therefore, we measured the Cu K-edge spectra of the complexes as well as two reference oxides: Cu_2_O and CuO. The copper K-edge corresponds to dipole-allowed 1s–4p transition and shifts to the higher energy values with increasing metal oxidation state. Figure 5 presents the comparison of Cu K-edge spectra of two complexes: **Cu2** (representative of alkylphenyltiourea derivatives) and **Cu4** (representative of halogenphenylthiourea complexes), and the reference oxides. The absorption edge for Cu_2_O is at 8979 eV and for CuO at 8982 eV. The energy positions and the shape of spectra of thiourea complexes are closer to CuO (Figure 5), which proves that analyzed complexes contain copper at a +2 oxidation state.

#### 2.2.4. EXAFS Studies

**Figure 6:**
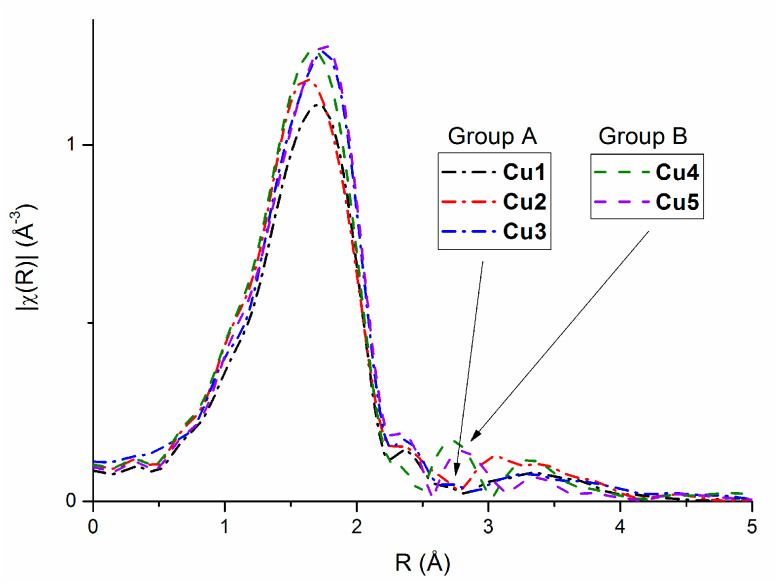
Fourier transformed experimental EXAFS oscillations for complexes.

Extended X-ray Absorption Fine Structure spectra were collected for all complexes: **Cu1**–**Cu5**. The comparison of Fourier-transformed experimental EXAFS oscillations (Figure 6), especially the region between 2.5 and 3 Å, indicates structural differences within the studied compounds. Therefore, the complexes were divided into two groups: A (**Cu1**–**Cu3**) without any peak between 2.5 and 3 Å and group B (**Cu4**, **Cu5**) with a peak in this region. 

**Figure 7:**
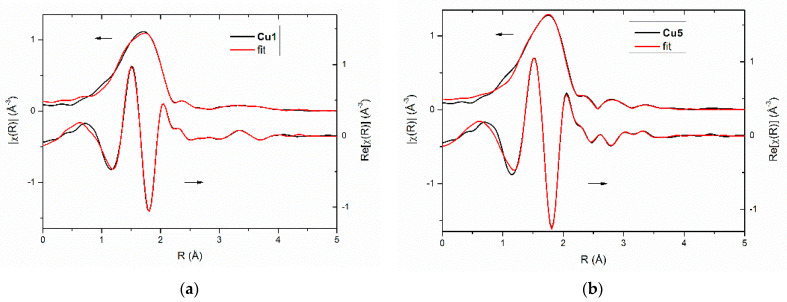
The best fits together with FT EXAFS oscillations of complexes: (**a**) **Cu1** and (**b**) **Cu5**.

The EXAFS analysis was performed in two steps. In the first step, several different models were checked. In this case, only single scattering paths were taken into account. Data obtained from the preliminary fitting (such as distances, and type and number of atoms) pointed out four similar models for group A and two for group B. Then, these models were checked and refined by DFT and XANES calculations (see next section). Finally, the EXAFS analysis of complexes was performed on the refined models and the multiple scattering paths were also used in the final fits. The best fits together with FT EXAFS oscillations for **Cu1**—representative of group A, and for **Cu5**—belonging to group B are presented in Figure 7 (fits for all complexes are presented in Figure S1 in Supplementary Materials). 

The study of complexes within group A indicates four atoms within the first coordination sphere (Table 2), two N and two S atoms. The Cu–N/S bond length is in the range 1.93–1.95 Å for nitrogen and 2.23–2.25 Å for sulfur atoms. Moreover, at a distance of around 2.5 Å, two carbon atoms were identified. 

For two other complexes (**Cu4**, **Cu5**), belonging to group B, the first coordination sphere is formed by two N and two S atoms, being at similar distances from Cu(II) cation as in the case of the previous group of compounds. However, in the next coordination sphere, the second copper ion at a distance of around 2.8 Å, as well as one S atom above 2.6 Å, were identified (Table 2). These results suggest a more complex dimeric structure of compounds belonging to group B. 

#### 2.2.5. Structural Models of Complexes—DFT and XANES Calculations

**Figure 8:**
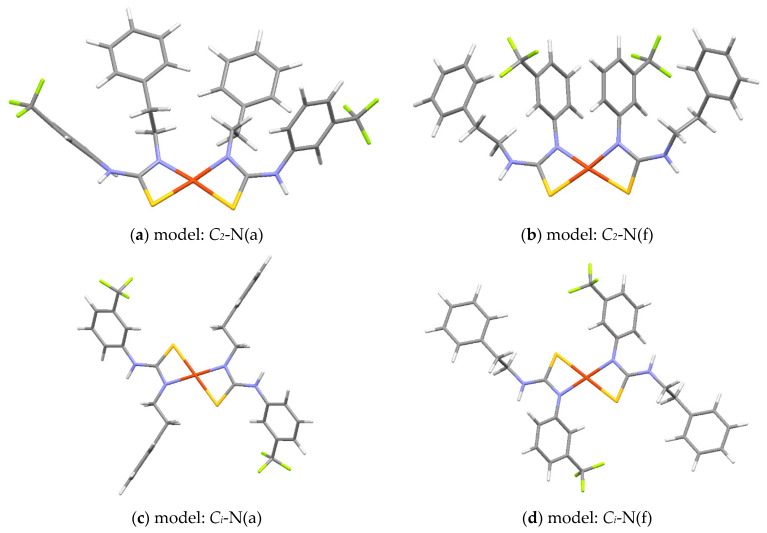
DFT optimized molecular structure of models of **Cu1** with CuN_2_S_2_ coordination sphere. The models are denoted by their point groups (*C_2_* and *C_i_*) and the type of N atom coordinating Cu: N(a) denotes nitrogen atom bond with the alkyl chain, and N(f) denotes nitrogen atom bonds with the trifluoromethylphenyl substituent.

**Figure 9:**
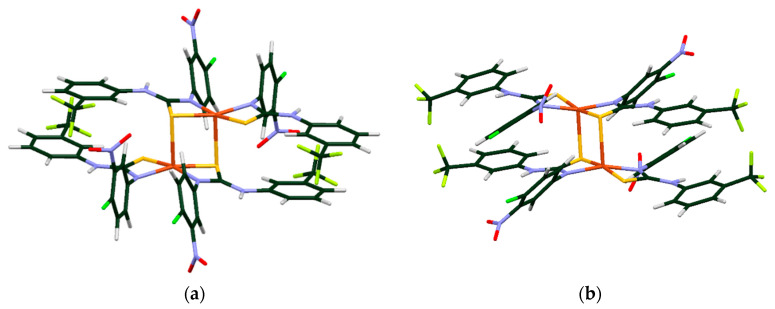
The structural dimeric models for representative **Cu5** complex composed of (**a**) noncentrosymmetric and (**b**) centrosymmetric monomers. The dimer as a whole belongs to the *C_i_* group.

On the basis of the spectroscopic data presented above, four possible structural models for complexes within group A (Figure 8) and two dinuclear models for complexes belonging to group B (Figure 9) were constructed and optimized. All relevant DFT structures are included in Supplementary Materials.

The structural models of complex **Cu1** (representative for group A) consist of two thiourea ligands coordinating via thiocarbonyl S and deprotonated N atoms to the Cu(II) cation, see Figure 8. These models differ in symmetry (*C_2_* or *C_i_*) and the N atom binds to the metallic center. The coordination through the N(f) atom attached to the trifluoromethylphenyl substituent (Figure 8b,d) is more likely, as such structures are over 40 kJ/mol more stable (Table 3) than the chelates where N(a) atom is attached to the alkyl chain (shown in the Figure 8a,c). 

All these models have Cu–N and Cu–S distances being about 2.0 and 2.3 Å, respectively (Table 3), which are in very good agreement with EXAFS fitting for the first coordination sphere (Table 2). Furthermore, the calculated Cu–C distances (around 2.6 Å) coincide with the experiment ones (Table 2), which additionally confirms the validity of the assumed models. 

Taking into account the EXAFS results for complexes belonging to group B, a sandwich dimer was found to be the most plausible structure. Two structural models of **Cu5** differing in symmetry of monomers (C_2_ or C_i_) were constructed and optimized (Figure 9). The calculation indicated that dimers composed of noncentrosymmetric monomers turned out to be more energetically stable (ΔE = 24 kJ/mol). The distance between both mononuclear complexes in DFT-optimized dimeric models is much larger than the values obtained from EXAFS data, i.e., equilibrium Cu–Cu distance amounts to 3.38 Å. However, if the dimer was optimized with a Cu–Cu distance fixed to the EXAFS value (2.68 Å), the resulting structure was only 10 kJ/mol less stable than the fully optimized model. This small energy difference is of the order of intermolecular interaction energy in the condensed phase, which implies that in solutions or solids such more compact dimers can be stabilized. 

**Figure 10:**
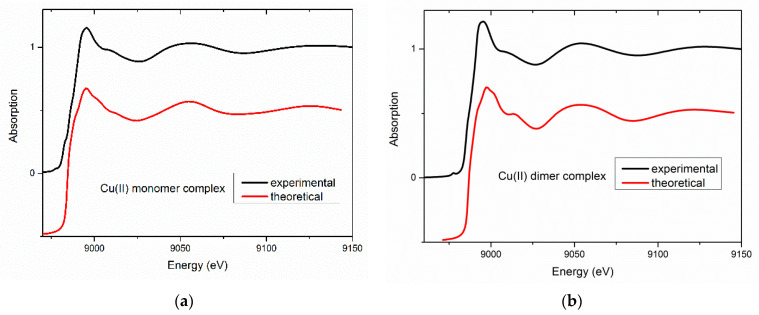
Calculated XANES spectra for the most energy-stable optimized models vs. experimental data for (**a**) **Cu1**—representative for monomeric alkylphenyl complexes (left) and for (**b**) **Cu5**—belonging to the dimeric complexes with halogenphenyl moiety.

In contrast to EXAFS, the shape of a XANES spectrum strongly depends on the angles between the neighboring atoms. The XANES spectra were measured for all complexes. Then, for two representative complexes from groups A and B (namely, for compounds **Cu1** and **Cu5**), the XANES spectra were calculated. The DFT-optimized structures were used as inputs; however, the distances between the absorbing and neighboring atoms were adjusted to the experimental values obtained from the EXAFS analysis. Comparison with the experimental XANES spectra (Figure 10) shows that the main features are reproduced confirming the validity of the proposed models.

### 2.3. Biological Evaluation

The newly synthesized complexes **Cu1**–**Cu5** were evaluated against a panel of standard and clinical bacterial strains, as well as towards mycobacteria. To establish the mechanism of bactericidal action, one complex was examined in both the topoisomerase IV decatenation assay and the DNA gyrase supercoiling assay. In addition, the cytotoxic effect of thiourea derivatives on different types of cancer cells was determined.

#### 2.3.1. In Vitro Antimicrobial Activity

The antimicrobial properties of title thiourea coordinates were examined against a broad series of microorganisms, using the method described before [16,17]. The investigation of standard bacterial strains revealed that the antibacterial potency varied from weak (compounds **Cu1**–**Cu3**) to considerable (for complex **Cu4**) and high (for **Cu5**) (Table 4). Alkylphenylthiourea complexes **Cu1**–**Cu3** inhibited the growth of staphylococcal isolates at the level of 16–64 µg/mL. However, derivatives with halogenphenyl moiety exerted markedly stronger antimicrobial activity, with MIC values from 8 µg/mL (complex **Cu4**) to 4 µg/mL (compound **Cu5**). 

The biological studies of (trifluoromethyl)phenylthiourea coordinates also included tests on therapeutically difficult clinical strains: methicillin-resistant *Staphylococcus aureus* (MRSA) and *Staphylococcus epidermidis* (MRSE), isolated from patients of clinical hospitals at the Medical University of Warsaw (Table 5). 

Against these bacterial strains, the complex of 4-chloro-3-nitrophenylthiourea **Cu5** was the most active. As the only one, it exerted very high potency for above half of tested *Staphylococci* (MIC = 2 µg/mL). A bit weaker, but still strong, it inhibited the growth of *S. aureus* 537, 585, and 586 strains, as well as half of the *S. epidermidis* isolates (MIC = 4 µg/mL). The compound **Cu5** showed higher effectiveness (up to 128 times) toward half of the clinical pathogens than the control antibiotic. The activity of other thiourea complexes against hospital staphylococcal strains was weak (derivatives **Cu1**, **Cu3**) to moderate (for **Cu2**, **Cu4**), and remained at the level of 8–128 µg/mL. Among the tested series, no relevant potency against Gram-negative strains of *E. coli* (MIC ≥ 128 µg/mL) and fungal species was detected. Although studied complexes are weaker antistaphylococcal agents than their starting ligands [20,21,22], the coordinate **Cu5** has retained the strong growth-inhibitory action against hospital isolates, as the corresponding unbound thiourea.

#### 2.3.2. Antitubercular Activity

The structural element of currently used tuberculostatic agents, e.g., isoxyl and its analogs, is the thiourea system combined with the flexible alkylaryl substituent. In this spirit, the evaluation of the in vitro antituberculosis activity of the synthesized derivatives was planned. The experiment was performed on the standard *M. tuberculosis* strain H_37_Rv and two “wild” strains isolated from patients suffering from tuberculosis: Spec 210 resistant to p-aminosalicylic acid (PAS), isoniazid (INH), ethambutol (ETB), and rifampicin (RMP), as well as the Spec. 192 strain, fully sensitive to the applied tuberculostatics. New complexes of 3-(trifluoromethyl)phenylthiourea showed weak activity toward H_37_Rv and Spec.192 strains; however, three compounds (**Cu2**, **Cu4**, and **Cu5**) strongly inhibited the growth of the resistant Spec. 210 isolate (Table 6). In relation to it, the inhibitory properties of the 3-bromophenyl- (**Cu4**) and 4-chloro-3-nitrophenylthiourea (**Cu5**) complexes were 4–8-fold stronger than the reference tuberculostatics. Additionally, the antitubercular potential of the molecule containing the 1-phenylethyl moiety (**Cu2**) was 2–4 times as strong as the control drugs. The other derivatives containing alkylphenyl moieties (**Cu1**, **Cu3**) reached the same or twice activity against Spec 210 as references. Due to the fact that the parental ligands **1**–**5** were not able to produce a noticeable growth inhibitory effect against the mentioned *M. tuberculosis* isolates [20,22], the complexation reaction improved their antimycobacterial profile. 

To eliminate potential carcinogens, complexes **Cu1**–**Cu5** were tested for DNA-damaging potency by rec-assay. For this aim, two genetically modified *Bacillus subtilis* strains were used: M45 (rec^−^; devoid of the recombinant-based DNA repair mechanism) and H17 (rec^+^), which is more sensitive to mutagenic substances than the H17 isolate. As shown in Table S1 (Supplementary Materials), no significant differences were found between the diameters of the inhibition zones denoted for both strains. This led to the conclusion that the tested thiourea complexes are non-genotoxic—they are not a source of genetic mutations. What is more, their biological action is not connected with a genotoxic activity.

#### 2.3.3. Type II Topoisomerase Inhibitory Activity

The mechanism of the antibacterial action of the most active complex of 4-chloro-4 nitrophenylthiourea (**Cu5**) was determined on a panel of bacterial topoisomerases including topoIV and DNA gyrase isolated from a *S. aureus* strain (Figures S2 and S3, Supplementary material). The obtained results exhibited that the antimicrobial activity of the investigated derivative is the result of a dual inhibitory ability against topoIV (IC_50_ = 6.20 μg/mL) and gyrase (IC_50_ = 16.80 μg/mL) (Table 7). The mentioned copper coordinate suppressed the activity of bacterial topoisomerase, and its antimicrobial profile is similar to other arylthioureas [20,22] and their metal coordinates [17] published by our group recently.

#### 2.3.4. Cytotoxicity

Since the metal-containing compounds possess promising anticancer properties [25,26,27,28], we established the cytotoxic effects of the **Cu1**–**Cu5** thiourea complexes. To determine the potential cytotoxicity of new compounds, the MTT assay was used. The complexes were tested against human carcinoma cell lines, such as SW480 (primary colon cancer), SW620 (metastatic colon cancer), and PC3 (metastatic prostate cancer), as well as against the normal cell line HaCaT (immortalized human keratinocytes). Table S2 (Supplementary Materials) gives the derivative concentrations that produced 50% of growth inhibition (IC_50_, μM), in contrast with two commonly used chemotherapeutics, doxorubicin, and cisplatin. 

**Figure 11:**
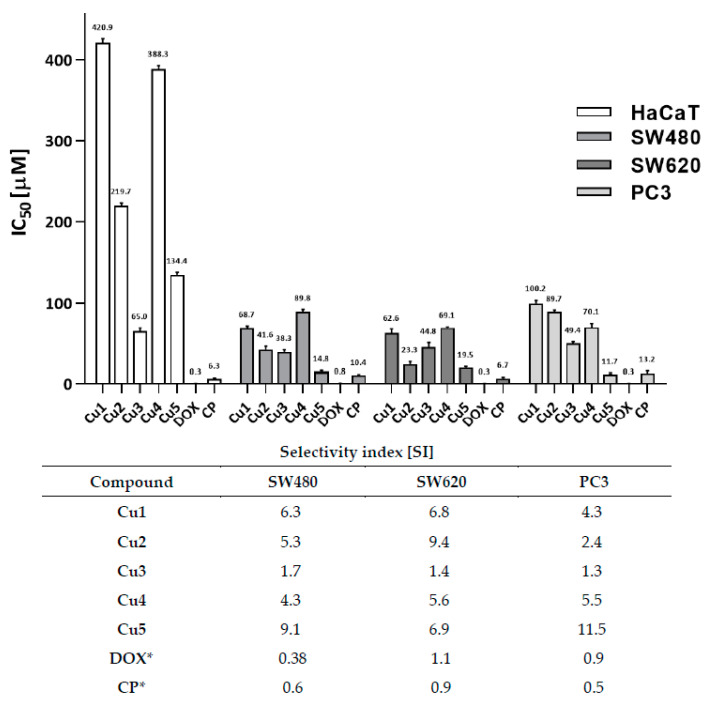
Cytotoxic activity (IC_50_, µM) of studied compounds estimated by the MTT assay. Bars are expressed as mean SD, IC_50_ (µM)—the concentration of the compound that corresponds to a 50% growth inhibition of the cell line (as compared to the control) after culturing the cells for 72 h with the individual compound. The SI (Selectivity Index) was calculated using the formula: SI = IC_50_ for normal cell line/IC_50_ cancer cell line. * The reference compounds: DOX—doxorubicin; CP—cisplatin.

The complexes exerted weak to moderate antitumor activity, simultaneously being non-toxic toward normal HaCaT cells (Figure 11). The most potent derivatives belonged to the group of phenylethyl (**Cu1, Cu2**) and 4-chloro-3-nitrothiourea (**Cu5**). Compounds **Cu1**, **Cu2**, **Cu4,** and **Cu5** were found to be up to severalfold less cytotoxic to the HaCaT cell line than the reference cytostatics. The highest selectivity (SI from 2.4 to 11.5) was observed for isomeric phenylethylthiourea coordinates **Cu1** and **Cu2** and halogenothiourea **Cu5**. The human primary (SW480) and metastatic (SW620) colon cancer cells appeared to be the most susceptible to the presence of the investigated arylthiourea derivatives—both in the aspect of IC_50_ (23.34 µM for the complex **Cu2**) and selectivity (SI = 6–16 observed for compounds **Cu1**–**Cu2**). The most promising halogen-containing compound **Cu5** has the lowest IC_50_ value towards all investigated cancer cells (11.7–19.5 µM), simultaneously with the highest selectivity versus HaCaT cells. That complex was also more effective against the PC3 cell line than cisplatin alone.

## 3. Materials and Methods

The chemicals were of analytical grade and were purchased from Sigma-Aldrich. The thiourea ligand was synthesized according to the procedure described elsewhere [20,21,22]. Elemental analyses were carried out on Vario Micro Cube. 

### 3.1. Synthesis 

The 1 mmol of thiourea ligand **1***–***5** was stirred in dimethylformamide (2 mL) until its dissolution; next 1 mmol of anhydrous copper(II) chloride was added to the prepared solution. The mixtures were stirred for 6 h at room temperature (approx. 21 °C). After evaporation of the solvent, the powder products were purified by washing thoroughly with water and dried at room temperature to yield complexes **Cu1***–***Cu5**. 

#### 3.1.1. Cu1, Copper(II) Complex with 1-(2-phenylethyl)-3-[3-(trifluoromethyl)phenyl]thiourea 

Quantities used were 0.32 g (1 mmol) **1** and 0.13 g (1 mmol) CuCl_2_ in DMF. Yield 54%; brown solid; Anal. Calc for ***Cu****(**1**)**_2_·H_2_O***: C,52.78; H, 4.20; N, 7.69 Found: C,52.67; H, 4.19; N, 7.75 (%).

#### 3.1.2. Cu2, Copper(II) Complex with 1-(1-phenylethyl)-3-[3-(trifluoromethyl)phenyl]thiourea

Quantities used were 0.32 g (1 mmol) **2** and 0.13 g (1 mmol) CuCl_2_ in DMF. Yield 50%; brown solid; Anal. Calc for ***Cu(2)_2_·0.75H_2_O***: C,53.10; H, 4.13; N, 7.74 Found: C,53.01; H, 4.16; N, 7.79 (%).

#### 3.1.3. Cu3, Copper(II) Complex with 1-benzyl-3-[3-(trifluoromethyl)phenyl]thiourea

Quantities used were 0.31 g (1 mmol) **3** and 0.13 g (1 mmol) CuCl_2_ in DMF. Yield 52%; green solid; Anal. Calc for ***Cu(3)_2_·0.5H_2_O***: C,52.13; H, 3.69; N, 8.11 Found: C,52.41; H, 3.71; N, 8.00 (%).

#### 3.1.4. Cu4, Copper(II) Complex with 1-(3-bromophenyl)-3-[3-(trifluoromethyl)phenyl] thiourea

Quantities used were 0.37 g (1 mmol) **4** and 0.13 g (1 mmol) CuCl_2_ in DMF. Yield 45%; green solid; Anal. Calc for ***Cu(4)_2_·0.5H_2_O***: C,40.97; H, 2.36; N, 6.82 Found: C, 40.75; H, 2.33; N, 6.88 (%). 

#### 3.1.5. Cu5, Copper(II) Complex with 1-(4-chloro-3-nitrophenyl)-3-[3-(trifluoromethyl) phenyl]thiourea

Quantities used were 0.37 g (1 mmol) **5** and 0.13 g (1 mmol) CuCl_2_ in DMF. Yield 41%; brown solid; Anal. Calc for ***Cu(5)_2_·H_2_O***: C,39.70; H, 2.16; N, 9.92 Found: C, 39.77; H, 2.15; N, 9.98 (%).

### 3.2. Spectroscopic Characterization

#### 3.2.1. ATR-IR Studies

Infrared spectra were performed on Nicolet iS5 (Thermo Scientific) FTIR Spectrometer with diamond ATR sample accessory. The thiourea complexes **Cu1**–**Cu5** as well as initial ligands **1**–**5** were recorded in the range of 400–4000 cm^−1^. 

#### 3.2.2. UV-Vis Studies

The electronic reflectance spectra of free ligands **1**–**5** and thiourea complexes **Cu1**–**Cu5** were collected on SHIMADZU UV-VIS Spectrophotometer UV-2600Plus with Integrating Sphere in the range of 220–1400 nm. 

#### 3.2.3. XANES Comparative Analysis

The K-edges of Cu were measured at the XAFS beamline in Elettra, Trieste, Italy. The spectra were recorded in the transmission mode. XANES spectra were collected for complexes **Cu2** and **Cu4,** and two reference oxides (Cu_2_O and CuO).

#### 3.2.4. EXAFS Studies

The EXAFS spectra were collected for all complexes at the XAFS beamline in Elettra, Trieste, Italy. The spectra were recorded in the transmission mode. For the XAS analysis, the Athena and Artemis programs included in the Demeter package [29,30] were used. The quantitative EXAFS analysis of complexes was performed as follows: k^2^ weighted χ(k) data were Fourier transformed in the k range 2.7 to 12 Å^−1^. The fitting was performed in the R space in the range from 1 to 4 Å. 

#### 3.2.5. Structural Models of Complexes—DFT and XANES Calculations

All structural models were optimized at DFT level using Perdew–Burke–Ehrzenoff exchange-correlation functional [31], as implemented in Turbomole code [32]. Gaussian basis sets from Ahlrich’s group were employed, namely, def2-TZVP [33] on Cu, S, and N atoms, whereas def-SV(P) [34] on the remaining ones. 

Theoretical ab initio XANES calculations were carried out using FEFF 9.6 code. The XANES, Self-Consistent Field, and Full Multiple Scattering cards were used. The Hedin–Lundqvist potential was chosen. 

### 3.3. Biological Evaluation 

#### 3.3.1. In Vitro Evaluation of Antimicrobial Activity 

The antimicrobial activity of the compounds was tested on Gram-positive bacteria (*Staphylococcus aureus* NCTC 4163, *Staphylococcus aureus* ATCC 25923, *Staphylococcus aureus* ATCC 6538, *Staphylococcus aureus* ATCC 29213, *Staphylococcus epidermidis* ATCC 12228, *Staphylococcus epidermidis* ATCC 35984); Gram-negative rods (*Escherichia coli* ATCC 10538, *Escherichia coli* ATCC 25922, *Pseudomonas aeruginosa* ATCC 15442, *Pseudomonas aeruginosa* ATCC 27863); and yeasts (*Candida albicans* ATCC 10231, *Candida albicans* ATCC 90028, *Candida parapsilosis* ATCC 22019). Hospital methicillin-resistant strains of *Staphylococcus aureus* and *Staphylococcus epidermidis* were obtained from the collection of the Department of Pharmaceutical Microbiology, Medical University of Warsaw, Poland. 

Minimal Inhibitory Concentration (MIC) was tested by the twofold serial microdilution method (in 96-well microtiter plates) using Mueller–Hinton Broth medium (Beckton Dickinson) for bacteria or RPMI-1640 medium for *Candida* species according to CLSI guidelines [35,36]. The stock solution of tested agent was prepared in DMSO and diluted in sterile water. Concentrations of tested agents ranged from 0.125 to 512 µg/mL. The final inoculum of all studied microorganisms was 10^5^ CFU/ mL^−1^ (colony-forming units per mL). Minimal inhibitory concentrations (the lowest concentration of a tested agent that prevents visible growth of a microorganism) were read after 18 h (bacteria) or 24 h (yeasts) of incubation at 35 °C. 

#### 3.3.2. Antitubercular Activity 

The synthesized compounds were examined in vitro for their tuberculostatic activity using MABA method (Microplate Alamar Blue Assay method) [37,38]. Investigations were performed by the twofold serial microdilution method (in 96-well microliter plates) using Middlebrook 7H9 Broth medium (Beckton Dickinson) containing 10% of OADC (Beckton Dickinson). The inoculum was prepared from fresh LJ culture in Middlebrook 7H9 Broth medium with OADC, adjusted to a no. 1 McFarland tube, and diluted 1:20. The stock solution of tested agent was prepared in DMSO. Each test compound stock solution was diluted in Middlebrook 7H9 Broth medium with OADC by fourfold, the final highest concentration to be tested. Compounds were diluted serially in sterile 96-well microtiter plates using 100 μL Middlebrook 7H9 Broth medium with OADC. Concentrations of tested agents ranged from 0.125 to 512 µg/mL. A growth control containing no antibiotic and a sterile control without inoculation were also prepared on each plate. The plates were incubated at 37 °C for a week. After the incubation period, 30 μL of Alamar blue solution was added to each well, and the plate was re-incubated for 24 h. Growth is indicated by a color change from blue to pink and the lowest concentration of compound that prevented the color change was noted as its MIC. Isoniazid (INH), Rifampicin (RMP), Streptomycin (SM), and Ethambutol (EMB) as reference drugs were used for comparison. 

##### Genotoxicity Studies

DNA-damaging activity of compounds was tested by *rec*-assay using two genetically modified *Bacillus subtilis* strains: M45 (rec^−^) and H17 (rec^+^) [39,40]. Tested compounds were dissolved in DMSO, and 10 µL of each solution was dripped onto sterile cotton discs (Rotilabo) to load 256 µg of a given compound per 9 mm disc. Discs were placed on the surface Nutrient agar plates (Difco) inoculated with 100 µL of bacterial overnight culture and incubated for 24 h at 35 °C. After incubation, the growth inhibition zones were measured. 4-Nitroquinoline N-oxide (NOQ) was used as reference genotoxin (concentration 2 µg per disc). Results of the genotoxicity test were estimated after 18 h of incubation at 35 °C by comparing the diameter of the inhibition zone on the *B. subtilis* M45 (rec^−^) strain with that on the *B. subtilis* H17 (rec^+^) strain. 

#### 3.3.3. Type II Topoisomerase Inhibitory Activity

##### *S. aureus* Topoisomerase IV Decatenation Assay 

The assay was performed using a *S. aureus* topoisomerase IV decantation kit (Inspiralis, Norwich, UK). Kinetoplast DNA (kDNA) was the substrate for topoisomerase IV. Firstly, 1 U of topoisomerase IV decatenated 200 ng of kDNA, in the dedicated decantation assay buffer supplied by the manufacturer. Enzyme activity was detected by incubation for 30 min at 37 °C in a total reaction volume of 30 µL and in the presence of different concentrations of the tested compound. The reactions were terminated by adding an equal volume of STEB buffer (40% sucrose, 100 mM Tris-HCl pH 8, 1 mM EDTA, 0.5 mg/mL bromophenol blue), followed by extraction with 1 volume of chloroform/isoamyl alcohol (24:1). Then, 20 µL of the aqueous phase of each sample was loaded onto a 1% agarose gel. Electrophoresis was conducted in Tris-acetate-EDTA buffer for 1.5 h at 80 V. Gels were stained with ethidium bromide and visualized under UV light in a transilluminator (ChemiDoc MP, Bio-Rad, Hercules, CA, USA). 

##### *S. aureus* DNA Gyrase Supercoiling Assay 

The assay was performed using a *S. aureus* DNA Gyrase Supercoiling kit (Inspiralis, Norwich, UK). The activity of DNA topoisomerase II was determined by measuring the conversion of relaxed pBR322 DNA to its supercoiled form. The reaction mixture contained 500 ng of mMpBR322 plasmid DNA and 1 U of enzyme and different concentrations of the compound in a total reaction volume of 30 µL. The mixture was incubated for 30 min at 37 °C. The reactions were terminated by adding an equal volume of STEB buffer (40% sucrose, 100 mM Tris-HCl pH 8, 1 mM EDTA, 0.5 mg/mL bromophenol blue), followed by extraction with 1 volume of chloroform/isoamyl alcohol (24:1). Then, 20 µL of the aqueous phase of each sample was loaded onto a 1% agarose gel. Electrophoresis was conducted in Tris-acetate-EDTA buffer for 3 h at 50 V. Gels were stained with ethidium bromide and visualized under UV light in a transilluminator (ChemiDoc MP, Bio-Rad, Hercules, CA, USA). 

#### 3.3.4. Cytotoxicity

The human primary (SW480), metastatic (SW620) colon cancer, metastatic prostate cancer (PC3), and human immortal keratinocyte (HaCaT) cell lines were purchased from the American Type Culture Collection (ATCC, Rockville, MD, USA). The cells were cultured in a medium according to protocols (MEM for SW480 and SW620, RPMI 1640 for PC3, and DMEM for HaCaT cells) supplemented with 10% fetal bovine serum (FBS), penicillin (100 U/mL), and streptomycin (100 μg/mL) and cultured in 37 °C/5% CO_2_ humidified incubator. The cells were cultured until appropriate confluence was achieved (80–90%). Next, they were harvested by treatment with 0.25% trypsin (Gibco BRL, San Francisco, CA, USA) and used for studies. 

To determine IC_50_ of the thiourea complexes, cells were seeded in 96-well plates (1 × 104 cells per well) and treated for 72 h with different concentrations of compounds. Cells without studied compounds in the medium were used as a control. 

The cell viability was assessed by determination of MTT salt (3-(4,5-dimethylthiazol-2-yl)-2,5-diphenyltetrazolium bromide) conversion by mitochondrial dehydrogenase. MTT assay was performed as previously described [41]. Experiments were repeated three times. Cell viability was presented as a percentage of MTT reduction in the treated cells versus the control cells. The number of viable cells cultured without studied compounds was assumed to be 100%. Decreased relative MTT level means decreased cell viability. Thiourea complexes with the highest cytotoxic potential assessed by MTT determination (with the lowest IC_50_) were chosen for subsequent assessments of cytotoxicity mechanisms.

## 4. Conclusions

The Cu(II) complexes with five 1,3-disubstituted thiourea derivatives were synthesized. All ligands contain 3-(trifluoromethyl)phenyl tail and differ in the second substituent. Three complexes contain an alkylphenyl group, the other two have a halogenphenyl part. The coordination compounds were structurally characterized by ATR-IR, UV-Vis, and XAFS spectroscopies. These studies indicated that copper is in the +2 oxidation state and revealed that the initial thioureas act as *N,S*-donor ligands (L). Upon the experimental studies, the structural models of complexes were proposed and optimized using molecular modeling. In these models, two thiourea ligands coordinate to the Cu(II) cation in bidentate fashion through thiocarbonyl S and deprotonated N atoms, thus forming a four-membered chelate ring with CuN_2_S_2_ moiety. All studied complexes are hydrates with the formula CuL_2_*xH_2_O. Such monomers are representative of alkylphenylthiourea complexes, whereas halogenphenyl derivatives formed sandwich-type dimers based on CuN_2_S_2_ units. Due to the continuous development of new theoretical and computational approaches to XANES, we decided to check its utility for the investigation of studied coordination complexes. Thus, the DFT structural models were used for XANES calculations. The theoretical XANES spectra agree with experimental ones, confirming the validity of the structures we have proposed. 

Parallel to the structural studies, the biological activity evaluation of new compounds was performed. The research carried out showed that alkylphenylthiourea complexes inhibited the growth of staphylococcal isolates at the level of 16–64 µg/mL, whereas derivatives with halogenphenyl moiety exerted markedly stronger antimicrobial activity, with MIC values from 4 to 8 µg/mL. Moreover, Cu(II) complex with a chloronitrophenyl part exerted very high potency for 19 of tested methicillin-resistant *Staphylococci* (MIC = 2 µg/mL). A similar relationship between biological activity and the structural characteristic was observed in the antitubercular profile of the studied complexes. The alkylphenylthiourea complexes showed weak activity against *Mycobacterium tuberculosis* strains with MIC values of 8–16 µg/mL, as opposed to the much better activity of halogenphenyl derivatives (MIC = 4 µg/mL). Simultaneously, all complexes showed no genotoxic effects. Moreover, the thiourea complex **Cu5** was found to be an effective inhibitor of both DNA gyrase and topoisomerase IV isolated from *Staphylococcus aureus*. 

The complexes exerted weak to fair antitumor activity (towards SW480, SW620, and PC3), simultaneously being non-toxic towards normal HaCaT cells. In addition to its antimicrobial profile, the complex **Cu5** was more potent and selective towards prostate cancer cells than the reference cisplatin.

## Figures and Tables

**Table 1:** The C = S stretching frequency in the infrared spectra of studied compounds.

νC = S (cm^−1^)					
Complex			Ligand		
** Cu1**	1300 (w)	764 (w)	** 1**	1351 (w)	844 (w)
** Cu2**	1301 (sh)	763 (w)	** 2**	1349 (w)	847 (w)
** Cu3**	1299 (sh)	764 (sh)	** 3**	1348 (w)	848 (w)
** Cu4**	1300 (w)	758 (w)	** 4**	1348 (w)	858 (w)
** Cu5**	1303 (sh)	754 (w)	** 5**	1342 (w)	854 (w)

**Table 2:** The distance from central cation to next atom R, number of atoms *n*, Debye–Waller factor *σ^2^* and *R*-factor of the fit evaluated during the final EXAFS fitting procedure. For clarity, only the main parameters for the closest neighbors are presented.

	Complex	Path	R (Å)	*N*	*σ^2^* (Å^2^)	*R*-Factor
**Group A**	** Cu1**	Cu–N	1.93(1)	2	0.005(1)	0.003
		Cu–S	2.23(1)	2	0.006(1)	
		Cu–C	2.53(1)	2	0.005(1)	
	** Cu2**	Cu–N	1.94(1)	2	0.005(1)	0.003
		Cu–S	2.23(1)	2	0.007(1)	
		Cu–C	2.57(1)	2	0.006(1)	
	** Cu3**	Cu–N	1.95(1)	2	0.005(1)	0.002
		Cu–S	2.25(1)	2	0.005(1)	
		Cu–C	2.58(1)	2	0.005(1)	
**Group B**	** Cu4**	Cu–N	1.93(1)	2	0.005(1)	0.004
		Cu–S	2.22(1)	2	0.006(1)	
		Cu–C	2.59(2)	2	0.005(1)	
		Cu–S	2.62(1)	1	0.006(1)	
		Cu–Cu	2.83(1)	1	0.006(1)	
	** Cu5**	Cu–N	1.95(1)	2	0.006(1)	0.004
		Cu–S	2.24(1)	2	0.005(1)	
		Cu–C	2.65(1)	2	0.006(1)	
		Cu–S	2.66(1)	1	0.005(1)	
		Cu–Cu	2.86(1)	1	0.008(1)	

**Table 3:** DFT geometry optimization results for considered mononuclear models of **Cu1** complex. The most energy-stable model is marked in bold.

Model of Complex Cu1		ΔE	R_Cu–N_	R_Cu–S_	R_Cu–C_
Symmetry	Coordinating Atom	(kJ/mol)	(Å)		
*C_2_*	N(a)	43	2.014	2.347	2.558
***C_2_***	**N(f)**	**0**	2.016	2.337	2.575
*C_i_*	N(a)	57	1.991	2.389	2.573
*C_i_*	N(f)	11	1.964	2.390	2.570

**Table 4:** In vitro activity of thiourea complexes **Cu1***–***Cu5** against standard bacterial and fungal strains—minimal inhibitory concentrations (MIC, μg/mL). Ref. *—Ciprofloxacin, Ref. **—Fluconazole.

	Cu1	Cu2	Cu3	Cu4	Cu5	Ref. *	Ref. **
*S. aureus* NCTC 4163	64	32	64	8	4	0.25	-
*S. aureus* ATCC 25923	64	32	64	8	4	0.5	-
*S. aureus* ATCC 6538	64	32	64	4	4	0.25	-
*S. aureus* ATCC 29213	64	32	64	8	4	0.25	-
*S. epidermidis* ATCC 12228	64	32	64	8	4	0.25	-
*S. epidermidis* ATCC 35984	64	16	64	8	4	≤0.125	-
*E. coli* NCTC 10538	≥256	≥256	≥256	128	>256	≤0.125	-
*E. coli* ATCC 25922	≥256	≥256	≥256	128	128	≤0.125	-
*P. aeruginosa* ATCC 15442	128	128	≥256	≥256	128	0.5	-
*P. aeruginosa* ATCC 27853	128	≥256	≥256	128	>256	0.5	-
*C. albicans* ATCC 10231	128	128	32	≥256	128	-	0.5
*C. albicans* ATCC 90028	128	128	32	≥256	128	-	0.5
*C. parapsilosis* ATCC 22019	64	128	32	≥256	128	-	0.5

**Table 5:** In vitro activity of thiourea complexes **Cu1***–***Cu5** against hospital methicillin-resistant strains of *Staphylococcus aureus* (MRSA) and *Staphylococcus epidermidis* (MRSE)—minimal inhibitory concentrations (MIC, µg/mL). Ref. *—Ciprofloxacin.

	Cu1	Cu2	Cu3	Cu4	Cu5	Ref. *
* S. aureus* 498	64	8	64	8	2	0.5
* S. aureus* 537	64	8	64	8	4	256
* S. aureus* 567	64	8	64	8	2	0.5
* S. aureus* 568	64	8	64	8	2	0.5
* S. aureus* 573	64	8	64	8	2	128
* S. aureus* 585	64	8	64	8	4	256
* S. aureus* 586	64	8	64	8	4	0.5
* S. aureus* 495	64	16	64	8	2	0.5
* S. aureus* 496	64	16	64	8	2	0.25
* S. aureus* 497	64	16	64	8	2	256
* S. aureus* 514	64	16	64	8	2	128
* S. aureus* 522	64	16	64	8	2	256
* S. aureus* 572	64	16	64	8	2	256
* S. aureus* 481	64	16	64	8	2	256
* S. epidermidis* 420	64	16	64	8	4	0.5
* S. epidermidis* 423	64	16	64	8	4	0.5
* S. epidermidis* 424	128	16	64	8	4	16
* S. epidermidis* 469	64	32	64	8	4	0.5
* S. epidermidis* 471	64	16	64	8	4	32
* S. epidermidis* 510	64	16	64	8	4	0.5
* S. epidermidis* 511	128	16	64	8	4	32
* S. epidermidis* 515	128	16	64	8	4	32
* S. epidermidis* 431	128	16	64	8	2	8
* S. epidermidis* 432	64	16	64	8	2	64
* S. epidermidis* 433	64	16	64	8	2	64
* S. epidermidis* 435	64	16	64	8	2	0.25
* S. epidermidis* 436	128	16	64	8	2	≤0.125
* S. epidermidis* 437	64	16	64	8	2	0.5
* S. epidermidis* 438	128	16	64	8	2	≤0.125
* S. epidermidis* 513	64	16	64	8	2	0.5

**Table 6:** Activity of complexes **Cu1***–***Cu5** against *Mycobacterium tuberculosis* strains—minimal inhibitory concentrations (MIC, µg/mL).

Compound	*M. tuberculosis* H_37_Rv	*M. tuberculosis* Spec. 192	*M. tuberculosis* Spec. 210
**Cu1**	16	16	16
**Cu2**	8	8	8
**Cu3**	16	16	16
**Cu4**	4	4	4
**Cu5**	4	4	4
Isoniazid (INH)	0.125	0.125	16
Rifampicin (RMP)	1	1	32
Streptomycin (SM)	1	1	16
Ethambutol (EMB)	2	2	32

**Table 7:** Inhibition of catalytic activities of *S. aureus* topoisomerases.

Compound	IC_50_ ^1^ ± S.E.M. for Topo IV (μg/mL)	IC_50_ ± S.E.M. for Gyrase (μg/mL)
Ciprofloxacin	1.70 ± 0.15	3.55 ± 0.13
**Cu5**	6.20 ± 0.50	16.80 ± 0.75

^1^ IC50—half of the maximal inhibitory concentration.

## Data Availability

All data generated or analyzed during this study are included in this published article; further inquiries can be directed to the corresponding author.

## Supplementary Materials

The supporting information can be downloaded at: https://www.mdpi.com/article/10.3390/ijms232415694/s1.
