# Supplementary material for: Synthesis, Structural Characterization and Biological Activity Evaluation of Novel Cu(II) Complexes with 3-(trifluoromethyl)phenylthiourea Derivatives
Source: Int J Mol Sci. 2022 Dec 10;23(24):15694. doi: 10.3390/ijms232415694 (PMC9779606; doi:10.3390/ijms232415694)
Supplement: Supplementary file 1 [file ijms-23-15694-s001.zip › ijms-2071859-Supplementary.pdf]

Supplementary Information

**Title: Synthesis, structural characterization and biological activity evaluation of novel Cu(II) complexes with 3-(trifluoromethyl)phenylthiourea derivatives**

**Figure S1**. The best fits together with FT EXAFS oscillations of complexes **Cu1-Cu5.**

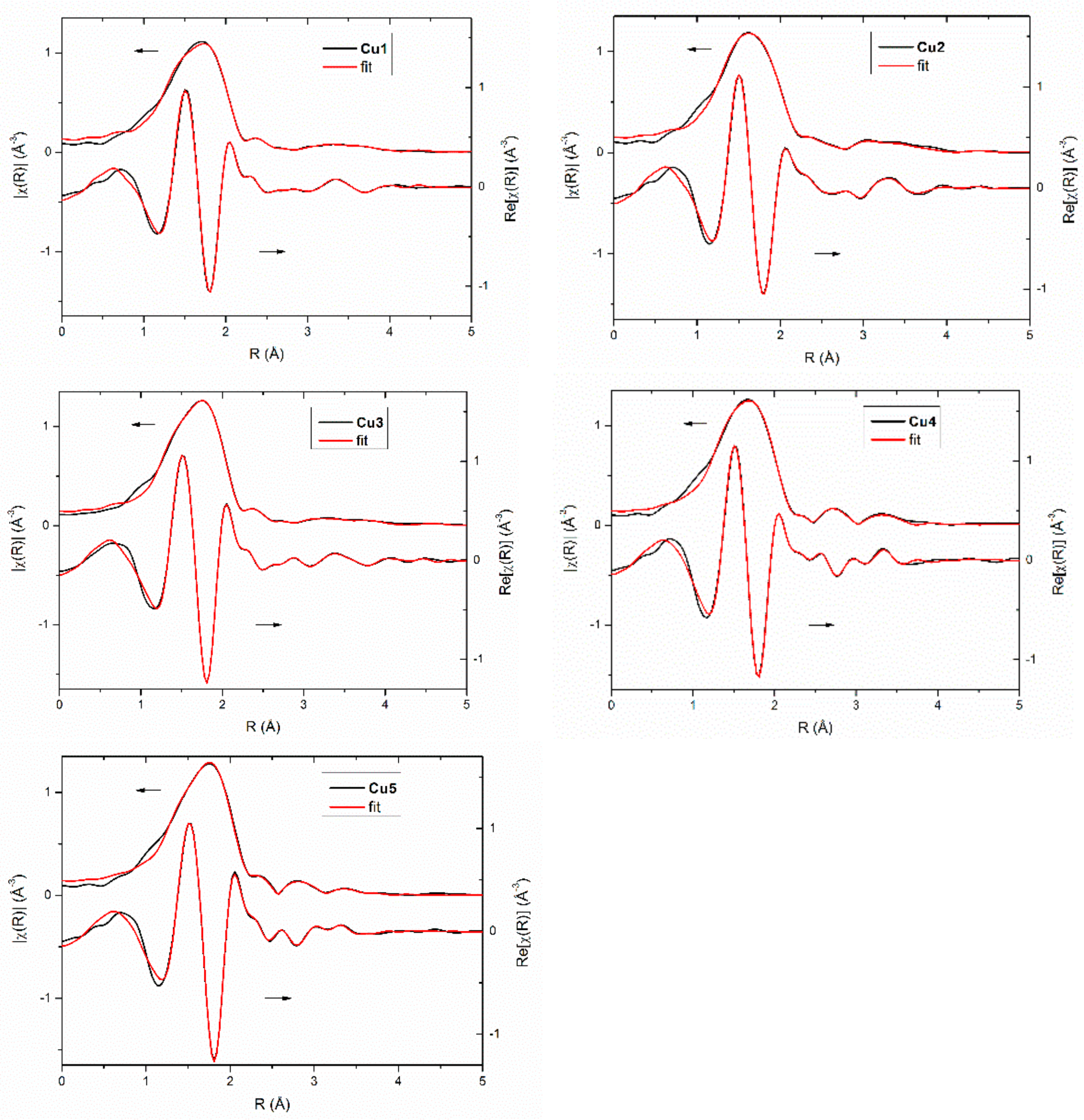

**Table S1.** Genotoxic activity of tested complexes – inhibition zone diameter (mm).

| Compound | *Bacillus subtilis strain* | |
|---|---|---|
| | **H17** | **M45** |
| **Cu1** | 16 | 16 |
| **Cu2** | 16 | 16 |
| **Cu3** | 15 | 15 |
| **Cu4** | 15 | 17 |
| **Cu5** | 18 | 17 |
| **NOQ** | 13 | 25 |

***NOQ** – 4-Nitroquinoline-N-oxide

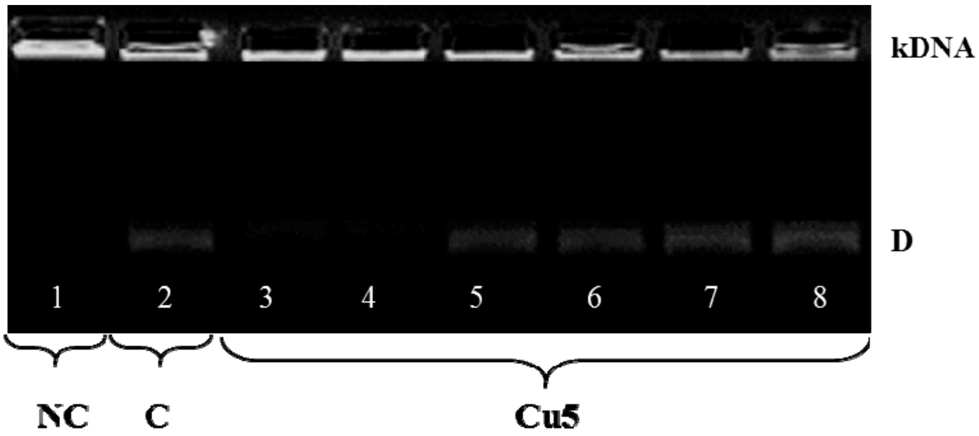


**Figure S2.** The effect of the complex **Cu5** on *S. aureus* topoisomerase IV activity assay showing catenated kDNA and the decatenated mini circles (D). Decreasing amounts of the compound was incubated with 200 ng kinetoplast DNA and run on agarose gel. Lane 1: incubation mixture without enzyme (negative control, NC). Lane 2: *S. aureus* DNA gyrase assay with dilution buffer (control, C). Lane 3-8: The complex **Cu5** at concentrations 32, 8, 4, 2, 1, 0.5 μg/ml, respectively.

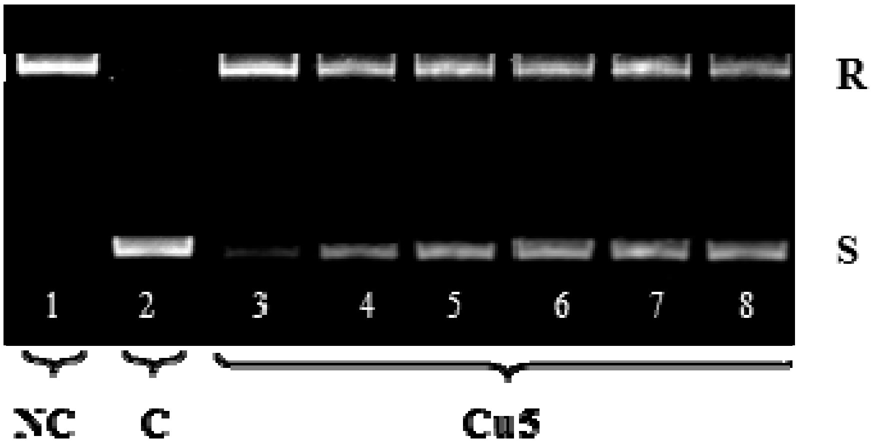


**Figure S3.** The influence of the complex **Cu5** on *S. aureus* DNA gyrase activity showing relaxed (R) and supercoiled DNA (S) bands. Decreasing amounts of the compound **Cu5** was incubated with supercoiled pBR322 plasmid DNA (500 ng) and run on agarose gel. Lane 1: incubation mixture without enzyme (negative control, NC). Lane 2: *S. aureus* DNA gyrase assay with dilution buffer (control, C). Lane: 3-8: The complex **Cu5** at concentrations 32, 8, 4, 2, 1, 0.5 μg/ml, respectively.

**Table S2.** Cytotoxic activity ($IC_{50}$, μM) of studied compounds estimated by the MTT assay[a].

| Compound | Cancer cells | | | | | | Normal cells |
|---|---|---|---|---|---|---|---|
| | **SW480[d]** | | **SW620[e]** | | **PC3[f]** | | **HaCaT[g]** |
| | $IC_{50}$[b] | SI[c] | $IC_{50}$ | SI | $IC_{50}$ | SI | $IC_{50}$ |
| **Cu1** | 68.7±3.4 | **6.3** | 62.6±4.9 | **6.8** | 100.2±3.6 | **4.3** | 420.9 ± 5.2 |
| **Cu2** | 41.58±4.3 | **5.3** | 23.34 ± 3.7 | **9.4** | 89.71±1.9 | **2.4** | 219.65 ± 3.4 |
| **Cu3** | 38.31 ±3.1 | 1.7 | 44.79± 5.6 | 1.4 | 49.4±2.4 | 1.3 | 64.98 ± 4.7 |
| **Cu4** | 89.82±2.6 | **4.3** | 69.07± 1.8 | **5.6** | 70.08±5.3 | **5.5** | 388.3 ± 4.4 |
| **Cu5** | 14.8±1.2 | **9.1** | 19.5±1.3 | **6.9** | 11.7±0.9 | **11.5** | 134.4±2.9 |
| Doxorubicin[h] | 0.75±0.1 | 0.38 | 0.26 ± 0.08 | 1.1 | 0.31±0.12 | 0.9 | 0.29 ± 0.11 |
| Cisplatin[i] | 10.40 ±0.90 | 0.60 | 6.70±1.10 | 0.90 | 13.20±2.10 | 0.50 | 6.30±0.7 |

[a] Data are expressed as mean SD, [b] $IC_{50}$ (μM) - the concentration of the compound that corresponds to a 50% growth inhibition of cell line (as compared to the control) after cultured the cells for 72 h with the individual compound. [c] The SI (Selectivity Index) was calculated using formula: SI = $IC_{50}$ for normal cell line/$IC_{50}$ cancer cell line. [d] Human primary colon cancer (SW480), [e] Human metastatic colon cancer (SW620), [f] Human metastatic prostate cancer (PC3), [g] Human immortal keratinocyte cell line from adult human skin (HaCaT). [h,i] The reference compound.
